# Identification of nectar sources foraged by female mosquitoes in Canada

**DOI:** 10.1093/jisesa/ieae033

**Published:** 2024-03-19

**Authors:** Bryan J Cassone, Ben G Pilling, Ana Borrego-Benjumea, Christophe M R LeMoine

**Affiliations:** Department of Biology, Brandon University, Brandon, MB R7A 6A9, Canada; Department of Biology, Brandon University, Brandon, MB R7A 6A9, Canada; Department of Biology, Brandon University, Brandon, MB R7A 6A9, Canada; Department of Biology, Brandon University, Brandon, MB R7A 6A9, Canada

**Keywords:** *Aedes*, *Culex*, soybean, nectar preferences, sugar feeding

## Abstract

For many mosquito species, the females must obtain vertebrate blood to complete a gonotrophic cycle. These blood meals are frequently supplemented by feeding on sugary plant nectar, which sustains energy reserves needed for flight, mating, and overall fitness. Our understanding of mosquito nectar foraging behaviors is mostly limited to laboratory experiments and direct field observations, with little research into natural mosquito-host plant relationships done in North America. In this study, we collected nectar-fed female mosquitoes over a 2-year period in Manitoba, Canada, and amplified a fragment of the chloroplast *rbcL* gene to identify the plant species fed upon. We found that mosquitoes foraged from diverse plant families (e.g., grasses, trees, ornamentals, and legumes), but preferred certain species, most notably soybean and Kentucky blue grass. Moreover, there appeared to be some associations between plant feeding preferences and mosquito species, date of collection, landscape, and geographical region. Overall, this study implemented DNA barcoding to identify nectar sources forage by mosquitoes in the Canadian Prairies.

## Introduction

Mosquitoes are ubiquitously found, medically important arthropod vectors of disease (Beerntsen et al. 2000). Their vector potential is rooted in a hematophagous lifestyle, as female mosquitoes acquire and transmit pathogens during host blood ingestion (Molaei et al. 2008, Melgarejo-Colmenares et al. 2022). Most species feed on the blood of vertebrates, which provides key nutrients that are required for egg production, including minerals, vitamins, and amino acids (Nasci 1984, Goldstrohm et al. 2003, Zhou et al. 2007). However, sugar feeding also represents an important source of nutrients for both sexes (Barredo and DeGennaro 2020). While males are obligate sugar feeders, females ingest plant sugar throughout their adult life, typically as floral and extrafloral nectar (Foster 1995, 2022). Sugar deprivation has been linked to reductions in energy reserves that can detrimentally impact the fecundity and survival of females (Foster 1995, Fernandes and Briegel 2005, Braks et al. 2006, Chadee et al. 2014). Indeed, plant nectar represents an important and often under-recognized aspect of the life history of female mosquitoes.

Research into the nectar sources foraged by mosquitoes is largely based on laboratory experiments and direct field observations of mosquito plant-feeding behaviors. Olfactory (Foster 1995, Gouagna et al. 2014), gustatory (Kessler et al. 2015), and visual (Peach et al. 2019) cues all appear to be utilized to detect/locate host plants, though the extent by which mosquitoes exhibit plant host specificity is not well established. A broad range of phytochemicals is attractive (i.e., act as semiochemicals) to diverse species, indicating that these dipterans are generalist plant feeders (Lothrop et al. 2012, Nyasembe et al. 2015, Steiner et al. 2018, Foster 2022, Hutcheson et al. 2022). However, many mosquitoes show an inherent preference for nectar-rich plants (Gouagna et al. 2014, Barredo and DeGennaro 2020), may feed disproportionately on particular species (Bowen 1992, Gadawski and Smith 1992, Junnila et al. 2010), and there is evidence of host discrimination based on lipid, glycogen, and protein content (Yu et al. 2018). Although not well studied, they may also become habituated to associating odorant stimuli (Jhumur et al. 2006, Sanford et al. 2013) and visual patterns (Bernáth et al. 2016) with sugar meals. Moreover, males and females show disparities in nectar preferences (Grimstad and DeFoliart 1974, Magnarelli 1979), presumably due to the sexual dimorphism in host plant feeding behaviors and accompanied physiological and metabolic differences (Foster 1995, Lomeli and Dahanukar 2022).

In the Canadian Prairies, *Aedes vexans* Meigen is the most commonly found species (Baril et al. 2023). It is a competent vector of California serogroup viruses (CSGVs), Rift Valley fever virus, West Nile virus (WNV), and Zika virus (Drebot 2015, Weissmann 2016, O’Donnell et al. 2017, Parry et al. 2020). *Ochlerotatus dorsalis* Meigen and *Culex tarsalis* Coquillett are also ubiquitous in the Prairies. Both are capable of transmitting Western equine encephalitis virus (WEEV), WNV, and CSGVs (Wood et al. 1979, Anderson et al. 2015). Other vector species that are less commonly found or associated with specific habitats include *Aedes canadensis* Theobald, *Coquillettidia perturbans* Walker, *Ochlerotatus triseriatus* Say, and *Ochlerotatus flavescens* Müller (Wood et al. 1979, Berry et al. 1986, McMahon et al. 2008, Anderson et al. 2015, Koloski et al. 2021).

DNA barcoding is a method that has been extensively used for species identification based on the sequence of a short, standardized genetic region (Ankola et al. 2021). In particular, the chloroplast ribulose diphosphate carboxylase (*rbcL*) gene is an effective barcode for plant species, as it is present in virtually all plant species and contains a region that is highly variable among species (CBOL Plant Working Group 2009, Bell et al. 2017). This approach and gene have been successfully employed to identify the plant/nectar content in the guts of diverse invertebrates (Matheson et al. 2007, Gravendeel et al. 2009, Junnila et al. 2011, Staudacher et al. 2011, Garcia-Robledo, et al. 2013, Kajtoch 2014, Lima et al. 2016, de Vere et al. 2017). To our knowledge, only 2 studies have used DNA barcoding to identify nectar sources in mosquitoes. Nyasembe et al. (2018) barcoded 29 *Aedes* and *Anopheles* specimens from Kenya using the *trnH-psbA* and *matK* genes, whereas Junnila et al. (2010) sequenced the *rbcL* of a small number of *Anopheles sergentii* (22) from Israel. Moreover, virtually nothing is known about the plant-feeding behaviors of mosquitoes in Canada. Consequently, we collected nectar-fed female mosquitoes over a 2-year period in Manitoba to characterize the nectar sources foraged by DNA analysis.

## Materials and Methods

### Mosquito Trapping

Collections were carried out between June and August over a 2-year period (2020 and 2021), which was previously described (Baril et al. 2023). In brief, CDC Miniature Light Traps (Model 1012, John W. Hock, Gainesville, FL) with carbon dioxide regulators set at 15 psi (light disabled) were used to sample host-seeking mosquitoes from dawn to dusk. Trapping was carried out twice weekly in 2020 and 2021, from June to August. We operated 24 traps in 8 West Manitoba communities in 2020, with 1 trap per community in 2021. Satellite traps from 9 additional locations in East Manitoba were provided to us by the City of Winnipeg Insect Control Branch. A description of all the sampling sites in which nectar-fed individuals were collected is displayed in Supplementary Table S1.

**Fig. 1.**
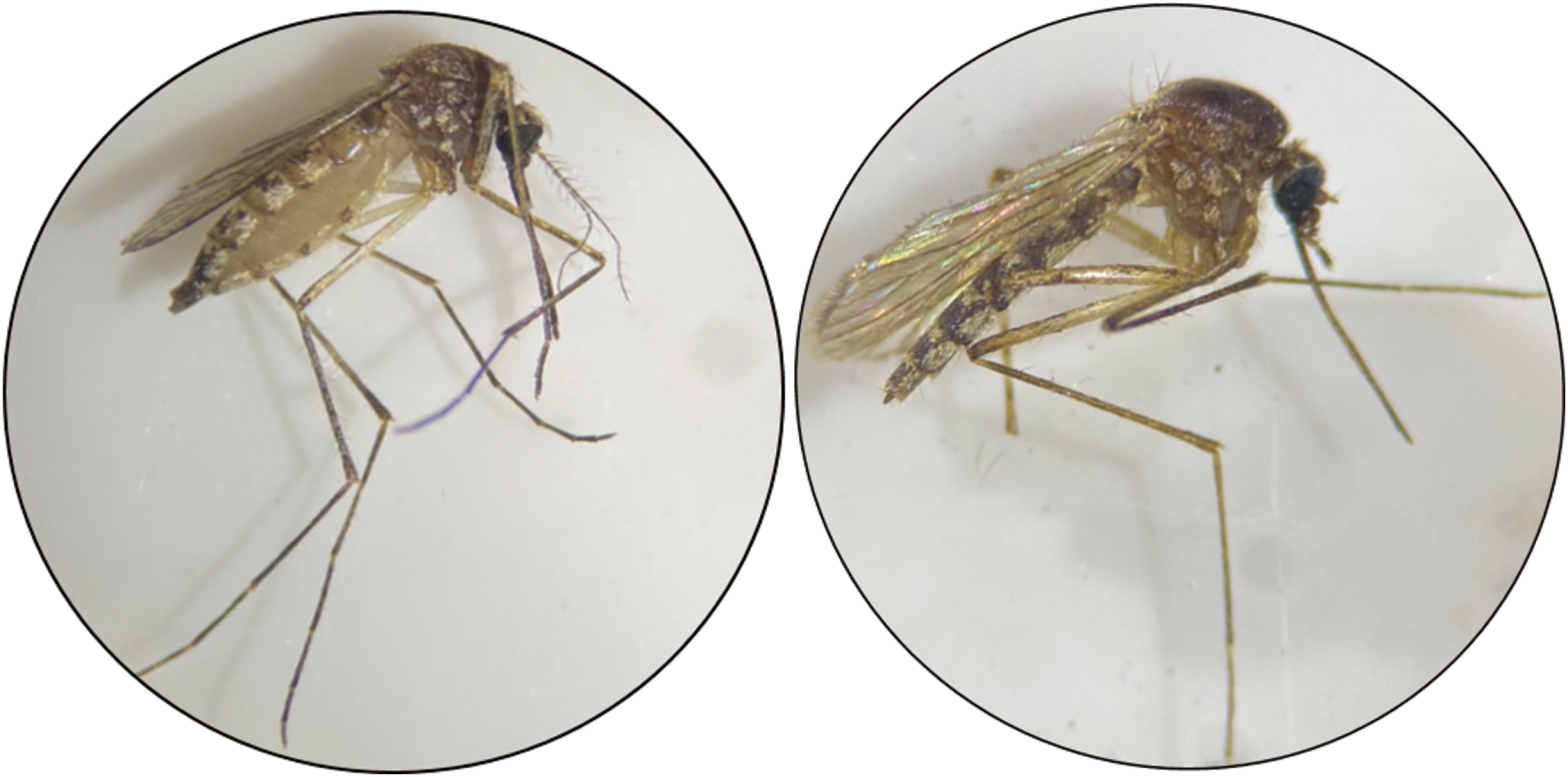
Representative nectar-fed (left) and non-fed (right) *Aedes vexans* females collected from Manitoba, Canada. The characteristically clear, distended abdomen can be readily visualized.

Nectar-fed individuals (i.e., possessing distended, clear abdomens; Fig. 1) were sorted out and identified to species using applicable mosquito identification keys (Carpenter and LaCasse 1955, Wood et al. 1979, Thielman and Hunter 2007). Specimens were then surface sterilized with 0.5% benzalkonium chloride followed by 70% ethanol and purified water (Yunik et al. 2015). Finally, each sample was placed in individual 1.5 ml tubes coded by species, date, and collection site, and stored at −80 °C until further processing.

### DNA Extraction, Sequencing, and Data Analysis

We first dissected out the abdomens (crop, guts) using a new set of sterilized scalpels and forceps for each nectar-fed specimen. The One-4-All Genomic DNA Miniprep Kit (Bio Basic, Markham, ON) was then used to isolate gDNA from nectar-fed individuals. We used the Nanophotometer NP80 (Implen Inc., Westlake Village, CA) to assess DNA quantity and quality. Amplification of the *rbcL* gene was carried out in 50 μl reactions using Phusion High-Fidelity PCR Master Mix and the following universal primer set (560 bp amplicon size): *rbcL*a-F: 5’-ATGTCACCACAAACAGAGACTAAAGC-3’ (Kress and Erickson 2007) and *rbcL*r506: 5’-AGGGGACGACCATACTTGTTCA-3’ (de Vere et al. 2012). Thermocycler (Biometra TOne, Analytik Jena, Germany) conditions consisted of 95 °C for 1 min followed by 35 cycles of 95 °C for 30 s, 51 °C for 30 s, and 68 °C for 1 min, with a final extension at 68 °C for 5 min (Bafeel et al. 2012). We visualized amplicons in 1% agarose gels stained with ethidium bromide using a ChemiDoc Imaging System (Bio-Rad Laboratories, Hercules, CA). In cases where the amplification was unsuccessful, the PCR reactions were redone using a different reverse primer: *rbcLa*-rev (600 bp amplicon size): 5’-GTAAAATCAAGTCCACCRCG-3’ (Kress et al. 2009) or *rbcLr*590 (590 bp amplicon size): 5’-AGTCCACCGCGTAGACATTCAT-3’ (de Vere et al. 2012). Amplicons were then sent to Génome Québec Innovation Centre (McGill University, Montreal, QC, Canada) for purification using a Biomek NX robot with a bead solution and Sanger sequencing of one or both the forward and reverse *rbcL*a strands using the 3730xl DNA Analyzer (Applied Biosystems, Waltham, MA). Resultant sequences were first visualized via their chromatogram and then identified to plant taxon (where possible) using BLASTn, tBLASTx, and the nr database at NCBI (https://blast.ncbi.nlm.nih.gov/Blast.cgi). Most sequences that could be resolved to the plant family had sequences similarities > 98% and query coverage of 100.

### Statistical Analysis

χ^2^ (goodness-of-fit) tests were first performed in R (R Core Team 2021) to explore differences in nectar preferences between communities, geographical regions, and collection dates. We computed *P* values by Monte Carlo simulation (100,000) with a threshold of significance of *P* < 0.05, no continuity correction, and an effective size (*w*) of 0.3. The null assumption is that the 2 categorical variables were independent. This analysis was not done between species due to the small sample sizes (<15) with the exception of *Ae. vexans*. Plant types with expected values of 0 were omitted from a given analysis.

## Results

A total of 265,564 mosquitoes were collected from our sampling sites throughout Manitoba, Canada over the 2-year trapping period. A very small number of collected specimens (*n* = 157) were nectar-fed and thus identified to the species and subjected to DNA barcoding. Of the subset of nectar-fed female mosquitoes, we successfully amplified a fragment of the *rbcL* gene from 135 specimens (West Manitoba = 75, East Manitoba = 60). The fluid collected from the ~14% that did not amplify may have consisted of water or non-sugar fluids rather than being derived from a plant source. Of those that successfully amplified, 85% (*n* = 115) generated sequence(s) that could be resolved to specific plant taxon. In nearly all cases, we were able to resolve the nectar source to the family level and to a lesser extent genus and species (Supplementary Table S2). A total of 19 plant families were identified, with Fabaceae (36%) and Poaceae (24%) being the most prevalent (Table 1). Within Fabaceae, 85% (*n* = 35) of the identified plants were soybean (*Glycine ma*x) and this also represented the most commonly foraged plant species overall (30% of the total). It was more challenging to resolve Poaceae to the species level, but based on the geographical location and sequence similarity, 34% (*n* = 13) of nectar sources identified from that plant family appeared to be Kentucky bluegrass (*Poa pratensis*). The remaining families detected represented ornamentals (17.4%), trees (12.2%), agricultural crops (7.8%), vines (1.7%), and wetland plants (0.9%). It should be noted that these plant types were subjective and there was some overlap among categories (e.g., soybean could be classified as both a legume and agricultural crop).

**Table 1.** Relative proportions of plant families fed upon by female mosquitoes. The nectar sources could be resolved to the family level for 115 specimens

Plant family	Description	Mosquitoes (%)
Fabaceae	** 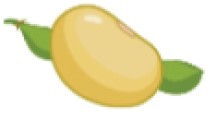 ** Legume	41 (35.7)
Poaceae	** 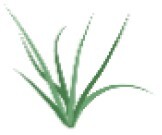 ** Grass	28 (24.3)
Musaceae	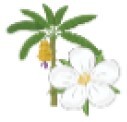 Ornamental	9 (7.8)
Solanaceae	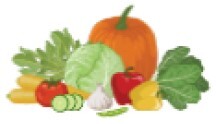 Agriculture	7 (6.1)
Asteraceae	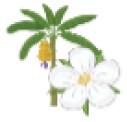 Ornamental	6 (5.2)
Pinaceae	** 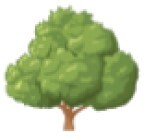 ** Tree	6 (5.2)
Oleaceae	** 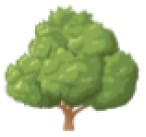 ** Tree	2 (1.7)
Salicaceae	** 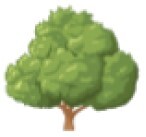 ** Tree	2 (1.7)
Smilacaceae	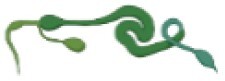 Vine	2 (1.7)
Ulmaceae	** 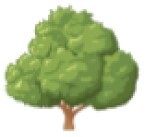 ** Tree	2 (1.7)
Amaranthaceae	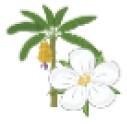 Ornamental	2 (1.7)
Adoxaceae	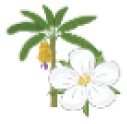 Ornamental	1 (0.9)
Apiaceae	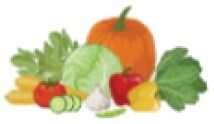 Agriculture	1 (0.9)
Brassicaceae	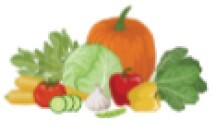 Agriculture	1 (0.9)
Geraniaceae	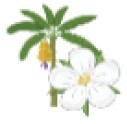 Ornamental	1 (0.9)
Juglandaceae	** 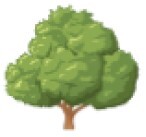 ** Tree	1 (0.9)
Menyanthaceae	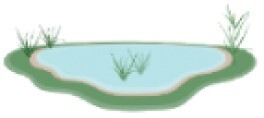 Wetland	1 (0.9)
Philadelpheae	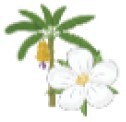 Ornamental	1 (0.9)
Sapindaceae	** 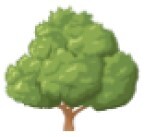 ** Tree	1 (0.9)

### Evidence of Nectar Preferences for Female Mosquitoes

**Fig. 2.**
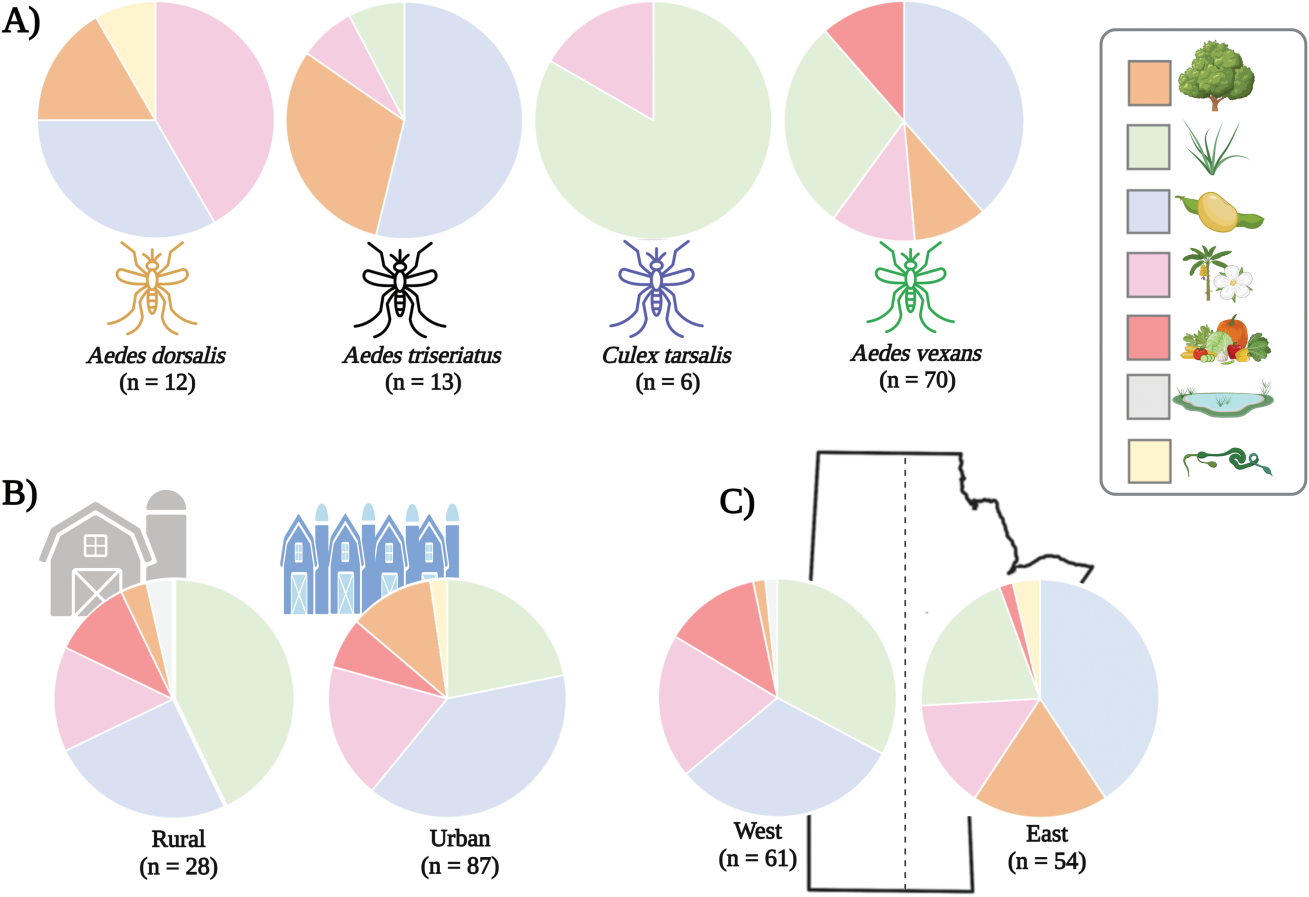
Partitioning of nectar sources in positive mosquitoes based on A) species, B) community (urban or rural), and C) region of the province (west or east). Each nectar source was first resolved to the plant family and then grouped to the most fitting plant type: legume, grass, ornamental, trees, agricultural crop, vine, or wetland plant. Significance was determined using χ^2^ goodness-of-fit tests, which indicated differences between communities and regions (*P* < 0.0001, both comparisons). We did not perform statistical analysis between species due to small sample sizes (<15) with the exception of *Aedes vexans*. The number of nectar-fed specimens for each contrast is represented in parentheses. This figure was created with Biorender.com (Science Suite Inc., Toronto, ON, Canada).

Figure 2 shows the partitioning of nectar sources in positive mosquitoes by species, community (urban or rural), and region of the province (west or east). Although we carried out χ^2^ tests on the dataset (with the exception of species), it should be emphasized that the relatively small and uneven sample sizes within most comparisons impacted the robustness of the analysis. The majority of nectar-fed mosquitoes captured were *Ae. vexans* (70), which primarily foraged on legumes and grasses (Fig. 2A). *Aedes dorsalis* predominately fed on ornamentals and legumes, *Aedes triseriatus* on legumes and trees, and *Cx. tarsalis* on grasses. In addition, there were significant differences in nectar preferences between communities (χ^2^ = 36.5, *df* = 5, *P* < 0.0001) and regions (χ^2^ = 60.4, *df* = 5, *P* < 0.0001). Mosquitoes captured within East Manitoba and/or urban areas were more likely to forage legumes and trees, whereas greater proportions of West Manitoba and/or rural mosquitoes were fed on grasses and agricultural crops (Fig. 2B and C). This was consistent when performing the regional statistical analysis with *Ae. vexans* as the only species included (χ^2^ = 64.04, *df* = 5, *P* < 0.0001). Finally, there were significant temporal differences in nectar-feeding behaviors (χ^2^ = 68.8, *df* = 4, *P* < 0.0001). In June, females foraged more agricultural crops and ornamentals and fewer trees and grasses than in July (Supplementary Figure S1).

## Discussion

The objective of our study was to provide insights into the nectar foraging behaviors of female mosquitoes in the Canadian Prairies. Our 2 consecutive years of trappings throughout southern Manitoba during the active season (June–August) yielded 135 nectar-fed females, which were subjected to DNA barcoding to identify the source plants. Laboratory experiments have uncovered possible sugar sources and preferences of mosquitoes (for review, see Foster 1995, Barredo and DeGennaro 2020); however, validation of these findings under natural conditions has almost exclusively relied on direct observations in the field (Foster 1995, 2022). One notable exception used DNA barcoding to provide clear and unbiased detection of the host plant preferences of *Anopheles sergenti* in the semi-desert south Jordan Valley of Israel (Junnila et al. 2010). No such studies have been undertaken in North America, and field investigations into natural mosquito-host plant relationships have been mostly limited to ground orchids and catchfly flowers (Stoutamire 1968, Thien and Utech 1970, Brantjes and Leemans 1976, Lahondère et al. 2020).

Our results suggest that female mosquitoes of the Canadian Prairies forage from a relatively small number of plant families that are varied (e.g., trees, grasses, ornamentals, and legumes). Although plants within Fabaceae and Poaceae represented the majority of the nectar sources, this is largely attributed to strong preferences for particular species within these families. Indeed, 42% of barcoded mosquitoes fed on nectar from soybean or Kentucky blue grass. This is consistent with field studies indicating that even mosquito species with diverse plant diets show preferential feeding on certain species (Sandholm and Price 1962, Grimstad and DeFoliart 1974, Magnarelli 1977, 1978, Gadawski and Smith 1992). Soybean fields represent an abundant nectar source, as a single plant produces 200–800 flowers (van Schaik and Probst 1958) and each flower can yield 0.5 µl of nectar with sugar concentrations up to 45% (Erickson 1984). To this end, the legume is a common and preferred nectar source of honeybees (*Apis mellifera* L.) in Midwestern United States of America (Lin et al. 2022). Soybean is widely cultivated in Manitoba, Canada, with more than 1.1 million acres sown in 2020 (SOY Canada 2020). This includes nearly 80% of legumes produced in the province. In our study, the ubiquitous use of soybean as a nectar source in both rural and urban areas was particularly surprising, given the latter collection sites were consistently > 2 km away from a field. Mosquito species in the genera *Culex* and *Aedes* typically have flight capacities above this threshold (Verdonschot and Besse-Lototskaya 2014) and their dispersal could be aided by wind (Service 1997). Nonetheless, there appears to be at least some degree of nectar preferences exhibited by Prairie mosquitoes for soybean. Feeding upon Kentucky blue grass may be indicative of more opportunistic foraging, as the grass is an aggressive invasive species found throughout the sampling region (Palit et al. 2021).

In addition to preferential feeding on certain plant species, several other notable trends could be teased out of our dataset. Most of the nectar-fed mosquitoes captured were *Ae. vexans*, which was expected given it is the most pervasive species in the Canadian Prairies (Brust and Ellis 1976, Baril et al. 2023). While the sample sizes were considerably smaller for the other barcoded species, it appears that their preferred nectar sources differed and may be associated with their life history. For instance, *Ae. triseriatus* had the highest proportion of sugar meals derived from trees. Colloquially known as the eastern tree hole mosquito due to its propensity to oviposit in standing water found in tree holes of hardwood forests (Barker et al. 2003, Koloski et al. 2021), its feeding preferences may represent opportunistic behaviors. Similarly, mosquitoes from West Manitoba foraged more grasses and agricultural crops than those from the East Manitoba, which seems to correspond to the ecoregions within the province (Smith et al. 1998). Future studies are needed that associate the mosquito nectar sources with characteristics of the flora surrounding the trapping site (e.g., plant species abundance, distance from the site, and average crown expansion) in order to better determine the extent by which mosquito plant feeding behaviors are opportunistic and selective.

There are other considerations pertaining to mosquito nectar foraging associated with our study. We collected and barcoded only female mosquitoes, and given the extensive metabolic and physiological differences between sexes (Foster 1995, Lomeli and Dahanukar 2022) it is plausible that they exhibit divergent plant feeding behaviors. There is some evidence that the sucrose:hexose ratio of a plant dictates sexual dimorphism, where males favor sucrose-rich nectar and females prefer hexose-rich nectar (Grimstad and DeFoliart 1974, Magnarelli 1979, Baker and Baker 1983). Future work is needed to determine whether differences exist between sexes in their preferred nectar sources. The capacity of mosquitoes to play important roles in plant pollination is also not fully established. Although there is considerable evidence of pollination by mosquitoes (e.g., Coleman 1934, Brantjes and Leemans 1976, Peach and Gries 2016, Lahondère et al. 2020), it is rarely investigated with unequivocal evidence (Foster 2022). Moreover, it is difficult to ascertain whether a given mosquito species is an essential pollinator for a specific plant species (Foster 2022). Soybean are self-pollinating plants, though biotic pollinators are capable of pollinating its flowers and increasing crop productivity to some extent (Milfont et al. 2013, Cunha et al. 2023). Kentucky blue grass is an aggressive invasive species that is predominately pollinated by wind and can in fact detrimentally impact pollinator diversity (Pei et al. 2023). Given the 2 commonly identified nectar sources do not rely on pollinators and others that are not intended to be pollinated (e.g., ornamentals), the contribution of mosquitoes to plant pollination may not be too significant, at least in the Canadian Prairies.

In conclusion, we characterized the plants fed upon by female mosquitoes in Manitoba, Canada via DNA barcoding. This represents some of the first DNA-based evidence of nectar feeding behaviors of mosquitoes in Canada. Female mosquitoes foraged from a relatively small number of plant families that are varied, with soybean as their preferred nectar source. Nevertheless, nectar-fed mosquito species appeared to have different foraging preferences, which may be influenced by a variety of factors (e.g., landscape and geographical region). Future research is needed to determine the extent by which these preferences are opportunistic/selective, differ between sexes, and play roles in plant pollination. For instance, semifield experiments with a selection of plants provided could better disentangle plant-specific preferences. Moreover, studies aimed at discerning the precise source of the plant fluids in barcoded individuals (e.g., floral nectar, extra-floral nectar, plant sap/phloem, and aphid honeydew) would also be of interest.

## Supplementary Material

ieae033_suppl_Supplementary_Tables_S1

ieae033_suppl_Supplementary_Tables_S2

ieae033_suppl_Supplementary_Figures_S1
