# Supplementary material for: Identification of nectar sources foraged by female mosquitoes in Canada
Source: J Insect Sci. 2024 Mar 19;24(2):11. doi: 10.1093/jisesa/ieae033 (PMC10949444; doi:10.1093/jisesa/ieae033)
Supplement: ieae033_suppl_Supplementary_Tables_S1 [file ieae033_suppl_supplementary_tables_s1.docx]

| City/town | Region | Community | Coordinates | Description |
| --- | --- | --- | --- | --- |
| Virden | West | Rural | 49.848668; -100.93253 | Adjacent a forest and horse riding area |
| Brandon-A | West | Urban | 49.823684, -99.962229 | Backyard in a residential area |
| Brandon-B | West | Urban | 49.831754, -99.966649 | Backyard in a residential area with some trees situated nearby |
| Brandon-C | West | Urban | 49.843075, -99.933283 | Backyard in a residential area, prominent gardens in this area |
| Cypress River | West | Rural | 49.556221; -99.090882 | Situated at forest edge, adjacent a swamp and field |
| Shoal Lake | West | Rural | 50.438087; -100.59073 | Next to a small lake and human dwellings |
| Souris | West | Rural | 49.620799; -100.25830 | Forest edge, close to residential area |
| Carberry | West | Rural | 49.867704; -99.360176 | Situated adjacent a forest and field |
| Killarney | West | Rural | 49.183215; -99.664190 | Close to town center, overlooking a field |
| Altona | East | Rural | 49.103498; -97.555495 | Field near the forest edge |
| Steinbach | East | Rural | 49.528528; -96.691092 | Forest edge, near a field with human dwelling in the distance |
| Winnipeg-A | East | Urban | 49.895077, -97.138451 | Front yard in residential area, trees situated throughout |
| Winnipeg-B | East | Urban | 49.838365, -97.081091 | Front yard in residential area |
| Winnipeg-C | East | Urban | 49.859025, -97.120644 | Residential area, field and sparse forest nearby |
| West St. Paul | East | Urban | 50.032371; -97.077923 | Adjacent human dwelling and forested area |

**Supplementary Table S1**. Description of the sampling sites nectar-fed mosquitoes were collected from. This includes the city/town, region, community, GPS coordinates, and a general description of the sampling site.
