# Supplementary figures and images for: Identification of nectar sources foraged by female mosquitoes in Canada
Source: J Insect Sci. 2024 Mar 19;24(2):11. doi: 10.1093/jisesa/ieae033 (PMC10949444; doi:10.1093/jisesa/ieae033)

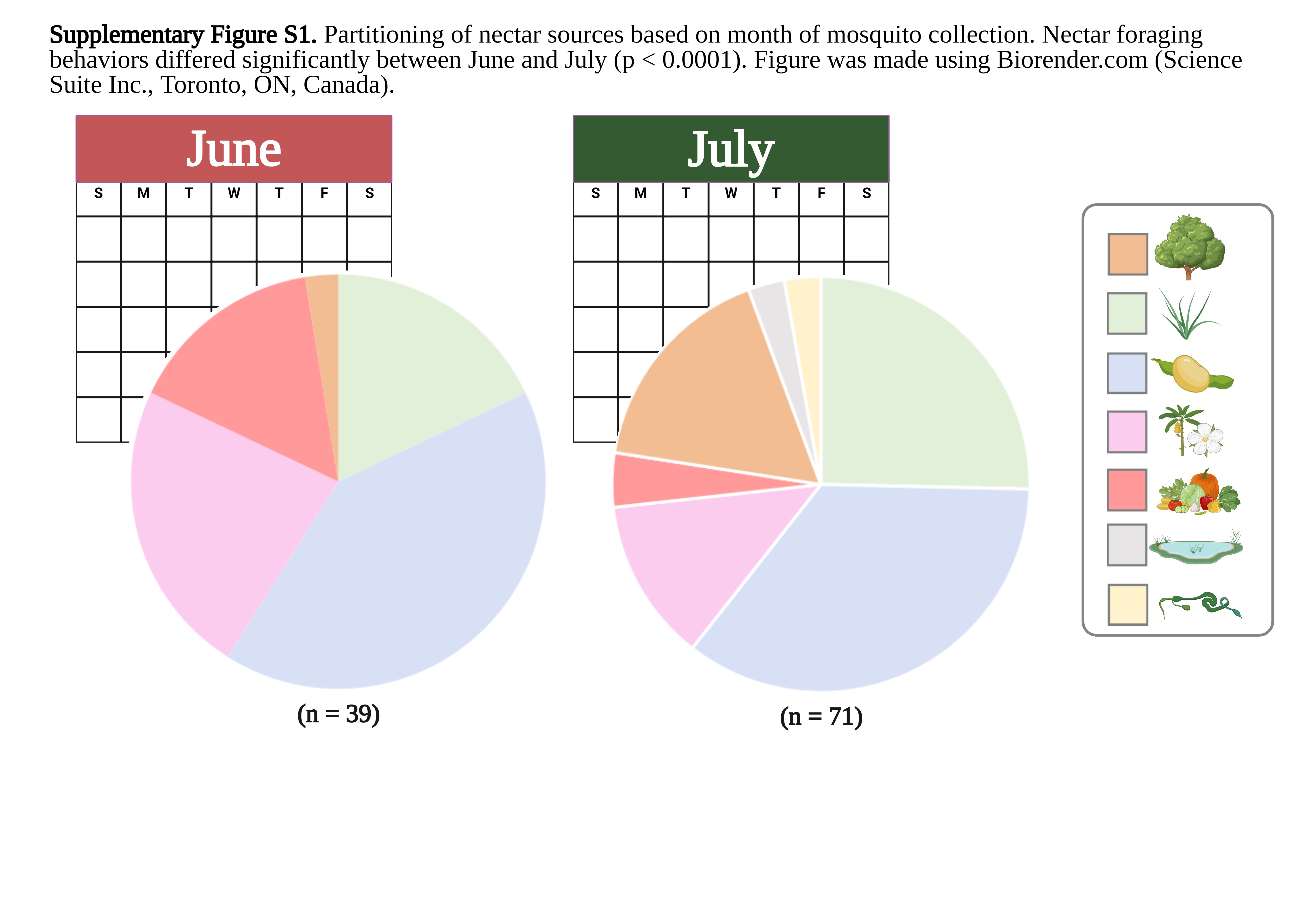

Supplement: ieae033_suppl_Supplementary_Figures_S1 [file ieae033_suppl_supplementary_figures_s1.jpeg]
